# Effect of the DGAT1 inhibitor pradigastat on triglyceride and apoB48 levels in patients with familial chylomicronemia syndrome

**DOI:** 10.1186/s12944-015-0006-5

**Published:** 2015-02-18

**Authors:** Charles Daniel Meyers, Karine Tremblay, Ahmed Amer, Jin Chen, Liewen Jiang, Daniel Gaudet

**Affiliations:** Novartis Institutes for Biomedical Research, Cambridge, MA USA; Department of Medicine, Université de Montréal and ECOGENE-21 Clinical Research Center, Chicoutimi, QC Canada; Novartis Institutes for Biomedical Research, East Hanover, NJ USA

**Keywords:** Lipoprotein lipase deficiency, Type 1 hyperlipoproteinemia, Orphan disease, LCQ908, Clinical trial, Pradigastat, Chylomicronemia, DGAT1

## Abstract

**Background:**

Familial chylomicronemia syndrome (FCS) is a rare lipid disease caused by complete lipoprotein lipase (LPL) deficiency resulting in fasting chylomicronemia and severe hypertriglyceridemia. Inhibition of diacylglycerol acyltransferase 1 (DGAT1), which mediates chylomicron triglyceride (TG) synthesis, is an attractive strategy to reduce TG levels in FCS. In this study we assessed the safety, tolerability and TG-lowering efficacy of the DGAT1 inhibitor pradigastat in patients with FCS.

**Methods:**

Six FCS patients were enrolled in an open-label clinical study. Following a 1-week very low fat diet run-in period patients underwent baseline lipid assessments, including a low fat meal tolerance test. Patients then underwent three consecutive 21 day treatment periods (pradigastat at 20, 40 & 10 mg, respectively). Treatment periods were separated by washout periods of ≥4 weeks. Fasting TG levels were assessed weekly through the treatment periods. Postprandial TGs, ApoB48 and lipoprotein lipid content were also monitored.

**Results:**

Following once daily oral dosing, steady-state exposure was reached by Day 14. There was an approximately dose proportional increase in pradigastat exposure at studied doses. Pradigastat was associated with a 41% (20 mg) and 70% (40 mg) reduction in fasting triglyceride over 21 days of treatment. The reduction in fasting TG was almost entirely accounted for by a reduction in chylomicron TG. Pradigastat treatment also led to substantial reductions in postprandial TG as well as apo48 (both fasting and postprandial). Pradigastat was safe and well tolerated, with only mild, transient gastrointestinal adverse events.

**Conclusion:**

The novel DGAT1 inhibitor pradigastat substantially reduces plasma TG levels in FCS patients, and may be a promising new treatment for this orphan disease.

**Trial registration:**

ClinicalTrials.gov identifier NCT01146522.

## Background

In response to a fat containing meal, chylomicrons (CM) are secreted by small intestine enterocytes into the bloodstream where they deliver dietary triglyceride (TG) to body tissues such as skeletal muscle and adipose. In most humans, CM-TG is rapidly cleared from the bloodstream by the enzyme lipoprotein lipase (LPL), which hydrolyzes the TG into free fatty acids, which are then taken up into the skeletal muscle or adipose for energy utilization or storage. Familial chylomicronemia syndrome (FCS), also known as type I hyperlipoproteinemia, is a rare autosomal recessive disease caused by loss-of-function mutations in the LPL gene or by mutations in genes encoding proteins or enzymes directly affecting LPL activity [1]. The complete loss of LPL activity significantly decreases the clearance of CM-TG, leading to CM accumulation in both the fed and fasted states (i.e. chylomicronemia), and severe hypertriglyceridemia [2,3].

Clinical signs and symptoms of FCS include recurrent episodes of abdominal pain, lactescent plasma, lipemia retinalis, skin lesions (eruptive xanthomas), hepatosplenomegaly, hematological disturbances, and occasionally spurious biochemical and hematological laboratory values [1]. Most importantly, FCS patients are at a very high risk of developing acute pancreatitis, which can be severe and life-threatening and is associated with high medical costs [4]. Chronic pancreatitis, pancreatic insufficiency, and cardiometabolic complications may also occur in patients with FCS [1]. Genetic LPL defects have also been associated with an increased risk of pulmonary embolism or atelectasia [5].

The primary therapeutic goal in FCS patients is to reduce hypertriglyceridemia to decrease the risk of acute pancreatitis. Unfortunately, currently available oral TG-lowering drugs which up-regulate LPL activity and/or down-regulate very-low-density lipoprotein (VLDL)-TG synthesis typically have little to no efficacy in FCS patients [6]. Without effective oral pharmacotherapy, patients are required to observe a very low fat diet (≤15% by calories) to lower their TG levels. Even with strict adherence to the very low fat diet, which is difficult to comply with for prolonged periods, TG levels are typically difficult to control [7]. LPL gene replacement therapy has recently been approved in Europe, although its use is currently restricted to the most severely affected patients with FCS due to an LPL gene defect and recurrent pancreatitis [8,9]. Its long-term efficacy is still under investigation.

Diacylglycerol acyltransferase-1 (DGAT1) catalyzes the final step in TG synthesis and is highly expressed in the small intestine enterocytes, where it plays a key role in absorption of dietary fat [10]. DGAT1-deficient mice are protected from the typical post-meal spike in plasma TG levels, and have significantly decreased post meal levels of CMs [11]. Based on this, DGAT1 inhibition is an attractive strategy to reduce the synthesis and secretion of CM-TG, and thereby lower total TG in FCS patients. Pradigastat (formerly LCQ908) is a potent and selective small-molecule DGAT1 inhibitor. Oral administration of pradigastat showed the median T_max_ of about 10 hours which slowly declined with a prolonged terminal elimination half-life of ~150 hours [12,13]. Pradigastat decreased CM-TG synthesis and secretion in animals resulting in blunted postprandial hypertriglyceridemia [14]. In healthy human volunteers, pradigastat decreased postprandial CM particle numbers and TG content, preventing postprandial hypertriglyceridemia, and therefore may be effective in lowering TG in patients with FCS [14].

The objective of this study was to assess the safety, tolerability, and effects of the DGAT1 inhibitor pradigastat on fasting and postprandial plasma TG in patients with FCS and severe hypertriglyceridemia.

## Results

### Patient characteristics and disposition

Six patients with genetically confirmed FCS were enrolled in the study (two males and four females, all Caucasian) with a mean (standard deviation [SD]) age of 51.5 (12.7) years. Mean height and weight of the patients were 165.0 (8.8) cm and 59.4 (15.8) kg, respectively, and mean body mass index was 21.5 (3.9) kg/m^2^. All the six patients completed treatment periods 1 and 2, while four patients completed period 3. Two patients withdrew from the study after period 2 for personal scheduling reasons. Individual demographic data is shown in Table 1.

**Table 1:** **Patient demographics**

	**Patient #1**	**Patient #2**	**Patient #3**	**Patient #4**	**Patient #5**	**Patient #6**	**Mean +/−SD**
Age (years)	66	35	53	38	63	54	51.5 +/− 12.7
Weight (kg)	36.5	77.7	74.2	57.7	47.0	63.1	59.4 +/− 15.8
Height (cm)	159	175	175	162	153	166	165.0 +/− 8.8
BMI (kg/m^2^)	14.4	25.4	24.2	22.0	20.0	22.9	21.5 +/− 3.9
Gender	Female	Male	Male	Female	Female	Female	-

All individuals were of the Caucasian race.

### Effects on plasma triglycerides

**Figure 1:**
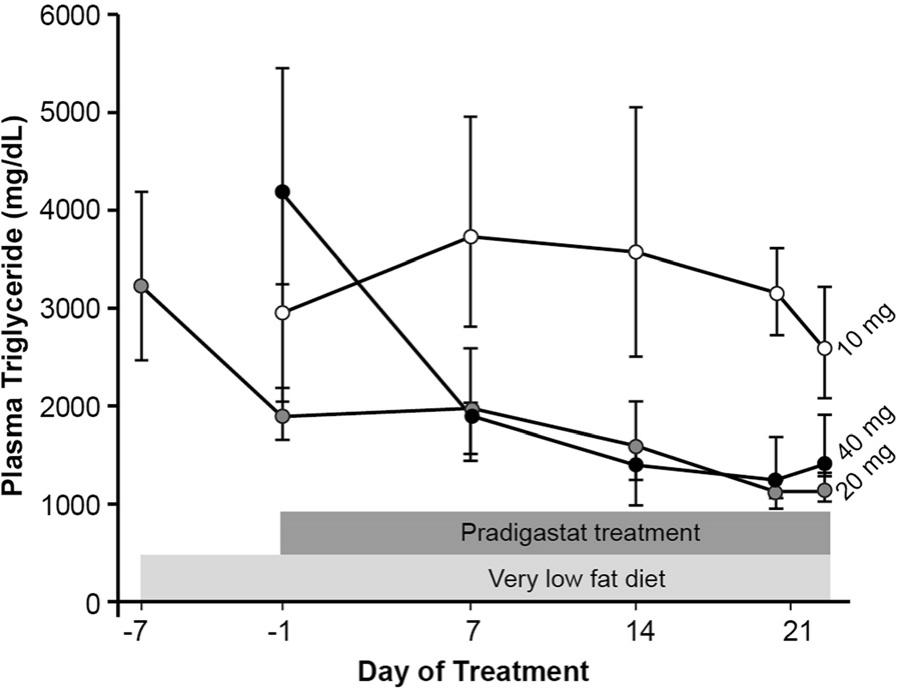
**Fasting triglyceride levels during the treatment with pradigastat at different doses.** Footnote: Data presented as geometric mean ± standard error of mean (SEM).

Changes in mean fasting TG levels during the treatment with pradigastat 10, 20, and 40 mg are shown in Table 2 and Figure 1. After 1-week dietary run-in period, geometric mean fasting TG level decreased from 3219.8 mg/dL (36.18 mmol/L; CV%, 71.7) on Day −7 to 1892.9 mg/dL (21.27 mmol/L; CV%, 31.8) on Day −1. Fasting TG level fell by an additional 41% from Day −1 at the end of 21 days’ therapy with pradigastat 20 mg (65% reduction from Day −7). During treatment period 2, there was a 70% reduction in fasting TG with pradigastat 40 mg at Day 21 compared with Day −1. No reduction in fasting TG was observed after 21-day treatment with pradigastat 10 mg during treatment period 3.

**Table 2:** **Effects of pradigastat on fasting and postprandial triglyceride and apolipoprotein B48Data presented are geometric means (CV %), unless otherwise specified**

	**Baseline** *	**Treatment period 1**		**Treatment period 2**		**Treatment period 3**	
		**Pradigastat 20 mg (n = 6)**		**Pradigastat 40 mg (n = 6)**		**Pradigastat 10 mg (n = 4)**	
		**EOT** ^**†**^	**Ratio** ^**‡**^	**EOT** ^**†**^	**Ratio** ^**‡**^	**EOT** ^**†**^	**Ratio** ^**‡**^
Fasting TG, mmol/L	19.21 (35.2)	12.80. (25.0)	0.67. (33.1)	13.97. (79.2)	0.73. (47.7)	32.14. (35.8)	1.41. (26.9)
Peak PPTG, mmol/L	20.91. (27.0)	12.91. (29.4)	0.62. (27.8)	14.38. (84.9)	0.69. (71.8)	41.22. (34.1)^§^	1.69. (37.3)^§^
AUC_0–9_ PPTG, h*mmol/L	163.49. (29.3)	103.16. (22.7)	0.63. (25.1)	114.69. (79.7)	0.70. (65.8)	321.68. (36.5)^§^	1.63. (37.9)^§^
Fasting ApoB48, g/L	0.071. (51.3)	0.063. (41.6)	0.88. (25.5)	0.043. (67.1)	0.61. (37.0)	0.113. (47.5)	1.29. (23.2)
Peak PP ApoB48, g/L	0.079. (34.1)	0.067. (39.7)	0.84. (23.8)	0.048. (56.8)	0.61. (28.6)	0.149. (29.7)^§^	1.53. (20.0)^§^
AUC_0–9_ PP ApoB48, h*g/L	0.632. (36.2)	0.527. (39.9)	0.83. (22.1)	0.378. (63.1)	0.60. (33.3)	1.261. (30.7)^§^	1.60. (16.2)^§^

ApoB48 = apolipoprotein B48; AUC_0–9_ = area under curve over 0 to 9 hours; CV = coefficient of variation; EOT = end of treatment; PP = postprandial; TG = triglycerides.

*Baseline values are at Day −1 for all the parameters, except for fasting TG and ApoB48 (mean of Day −1 and Day 1); ^**†**^EOT values are at Day 21 for all parameters, except for fasting TG and ApoB48 (mean of Day 21 and Day 22); ^‡^Geometric mean ratio to baseline; ^§^n = 3.

Two patients receiving pradigastat 20 mg (n = 6) and three patients receiving pradigastat 40 mg (n = 6) had attained end-of-treatment fasting TG levels below 1000 mg/dL. Furthermore, four patients had ≥30% reduction in fasting TG, while one patient had ≥50% reduction after receiving pradigastat 20 mg. Reduction in fasting TG with the 20 and 40 mg doses was statistically significantly greater than that with the 10 mg dose (ratio of geometric LS [Least Squares] mean ratio to baseline, 0.49 [90% confidence intervals [CI] 0.34–0.71] and 0.51 [90% CI 0.35–0.75], respectively; *p* < 0.01 for both). The TG-lowering effects of the 20 and 40 mg doses were not significantly different from each other (1.04 [90% CI 0.75–1.44], *p* = 0.83).

**Figure 2:**
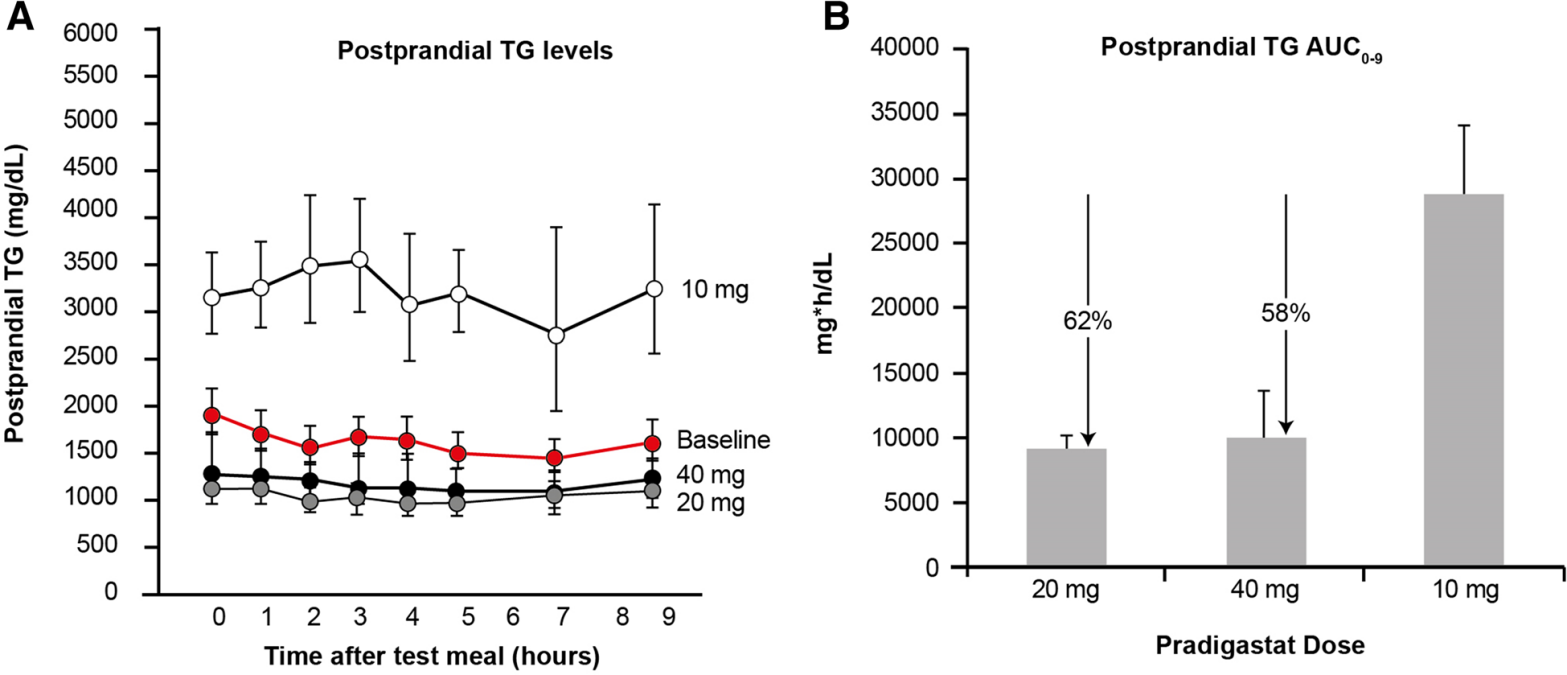
**Postprandial plasma triglyceride levels during the treatment with pradigastat at different doses.** Footnote: Data presented as geometric mean ± standard error of mean (SEM). Percentage change compared with the 10 mg dose for postprandial TG AUC_0–9_ are shown by ‘↓’. TG, triglycerides; AUC_0–9_, area under curve over 0 to 9 hours of meal tolerance test. Panel **A** = TG concentration/time profile over 9 hours, Panel **B** = TG AUC0-9.

Changes in postprandial plasma TG levels during the study are shown in Table 2. The baseline meal tolerance test (MTT) led to TG levels of around ≈ 2000 mg/dL (≈20 mmol/L) over 9 hours, with a slight increase in TG levels between 2 and 6 hours. Overall, postprandial TG during the entire MTT duration was higher with pradigastat 10 mg compared to baseline MTT (Figure 2A). Both peak postprandial TG value (−38%) and area under the curve over the 9 hours (AUC_0–9_; −37%) were reduced with pradigastat 20 mg. Reduction in peak postprandial TG (−31%) and AUC_0–9_ (−30%) was also noted with pradigastat 40 mg. Both the 20 and 40 mg doses led to significantly lower AUC_0–9_ (Figure 2B) than the 10 mg dose (ratio of geometric LS mean ratio to baseline, 0.38 [90% CI 0.22–0.67] and 0.42 [90% CI 0.24–0.74], respectively; *p* < 0.022 for both). Furthermore, three patients each had ≥30% reduction in peak postprandial TG and AUC_0–9_ and two patients each had ≥50% reduction in the same parameters with pradigastat 20 mg on reduction response assessment.

### Effects on apolipoprotein B48

**Figure 3:**
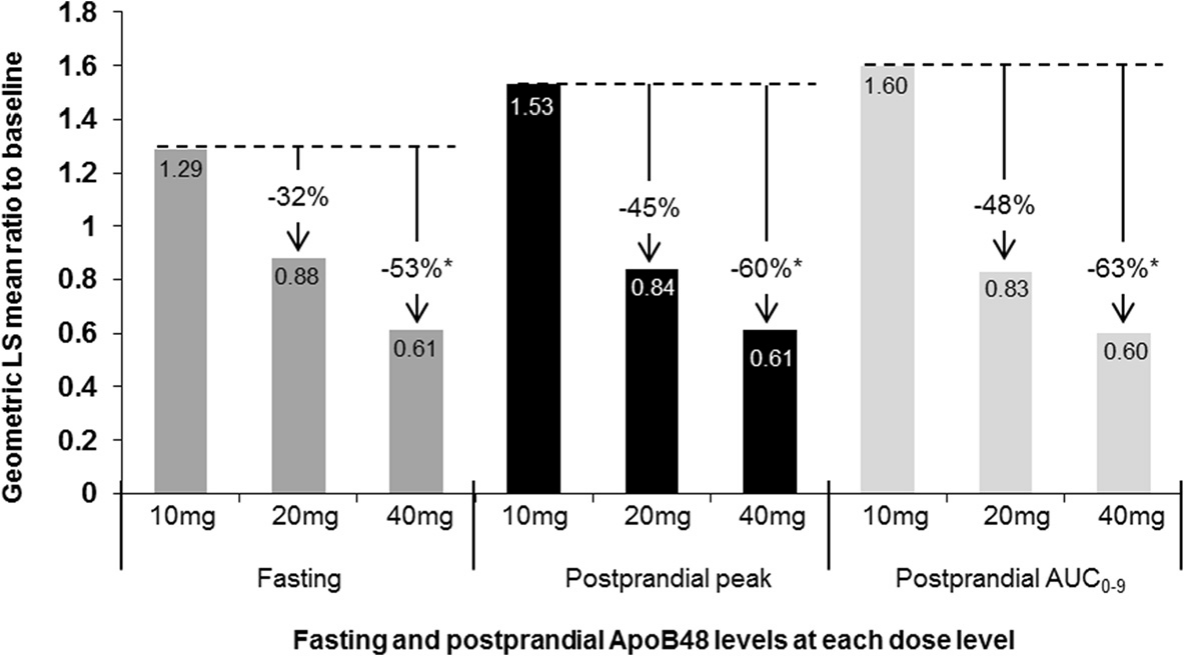
**Dose comparison of fasting and postprandial apolipoprotein B48 levels.** Footnote: Original ApoB48 values were expressed in ng/L for fasting and postprandial peak, and h*ng/mL for postprandial AUC_0–9_. This figure shows the Geometric least squares mean ratio of the end of treatment for each of the three dose levels to baseline. Percentage change compared with the 10 mg dose are shown by ‘↓’; * *p* < 0.05.

Both fasting and postprandial plasma apolipoprotein (ApoB48) levels decreased after treatment with pradigastat at 20 and 40 mg doses (Table 2). Compared with the 10 mg dose, both 20 mg (ratio of geometric LS mean ratio to baseline, 0.67 [90% CI 0.49–0.93], *p* = 0.051) and 40 mg (0.46 [90% CI 0.34–0.64], *p* = 0.002) doses were associated with a significant decrease in fasting ApoB48. Compared with the 20 mg dose, the 40 mg dose of pradigastat had a significant reduction in fasting ApoB48 (0.69 [90% CI 0.52–0.91], *p* = 0.035). In addition, both the 20 and 40 mg doses significantly decreased the postprandial peak and AUC_0–9_ ApoB48 levels compared with the 10 mg dose (Figure 3).

### Effects on lipoprotein fractions and other parameters

Effects of pradigastat on lipoprotein fractions as arithmetic means and the corresponding % change from baseline are shown in Table 3. As expected, CMs accounted for the majority of baseline total plasma TG (≈90%) and cholesterol (≈75%). Compared with each treatment period’s baseline, CM-TG content was consistently reduced at all the dose levels. Furthermore, CM cholesterol content was also reduced, with incremental reductions on higher doses. Pradigastat had mixed effects on low-density lipoprotein (LDL) and VLDL lipid content. Even with a modest increase in LDL-C seen at the 20 mg dose, the absolute post-treatment LDL-C was low (43.3 mg/dL). Pradigastat treatment generally increased HDL lipid content (cholesterol in particular).

**Table 3:** **Mean change in lipid content of lipoprotein fractions with pradigastat**

	**Treatment period 1**			**Treatment period 2**			**Treatment period 3**		
	**Pradigastat 20 mg (n = 6)**			**Pradigastat 40 mg (n = 6)**			**Pradigastat 10 mg (n = 4)**		
	**Baseline**	**EOT**	**% Change**	**Baseline**	**EOT**	**% Change**	**Baseline**	**EOT**	**% Change**
**CM-TG**	22.11. (7.51)	8.95. (1.86)	−59.5%	51.97. (49.78)	12.64. (7.12)	−75.7%	52.90. (10.11)	30.93. (13.48)	−41.5%
**CM-C**	5.38. (2.20)	3.32. (0.89)	−38.3%	9.50. (6.28)	4.38. (3.23)	−53.9%	8.48. (2.91)	6.45. (3.46)	−23.9%
**LDL-TG**	0.65. (0.22)	0.55. (0.21)	−15.4%	1.33. (2.19)	0.36. (0.17)	−72.9%	0.65 (0.17)	0.50. (0.29)	−23.1%
**LDL-C**	0.99. (0.34)	1.10. (0.25)	11.1%	1.01. (0.40)	1.02. (0.26)	1.0%	0.93. (0.39)	0.79 (0.19)	−15.1%
**HDL-TG**	0.25. (0.1)	0.27. (0.1)	8.0%	0.25. (0.10)	0.22. (0.04)	−12.0%	0.20. (0.08)	0.25. (0.06)	25.0%
**HDL-C**	0.34. (0.12)	0.55. (0.09)	61.7%	0.14. (0.13)	0.46. (0.11)	228.6%	0.17. (0.21)	0.25. (0.14)	47.1%
**VLDL-TG**	2.75. (0.24)	2.40. (0.45)	−12.7%	2.52. (1.45)	3.06. (0.75)	21.4%	3.55. (1.53)	3.53. (1.62)	−0.6%
**VLDL-C**	1.10. (0.56)	1.08. (0.28)	−1.8%	0.51. (0.59)	1.40. (0.55)	63.6%	0.23. (0.25)	0.94. (0.45)	308.7%

Data presented in mmol/L as arithmetic mean (SD) unless otherwise specified.

C = cholesterol; CM = chylomicrons; EOT = end of treatment; HDL = high-density lipoprotein; LDL = low-density lipoprotein; TG = triglycerides; VLDL = very-low-density lipoprotein.

Arithmetic means are presented since geometric means could not be calculated for all parameters due to zero values.

### Pharmacokinetic assessment

**Figure 4:**
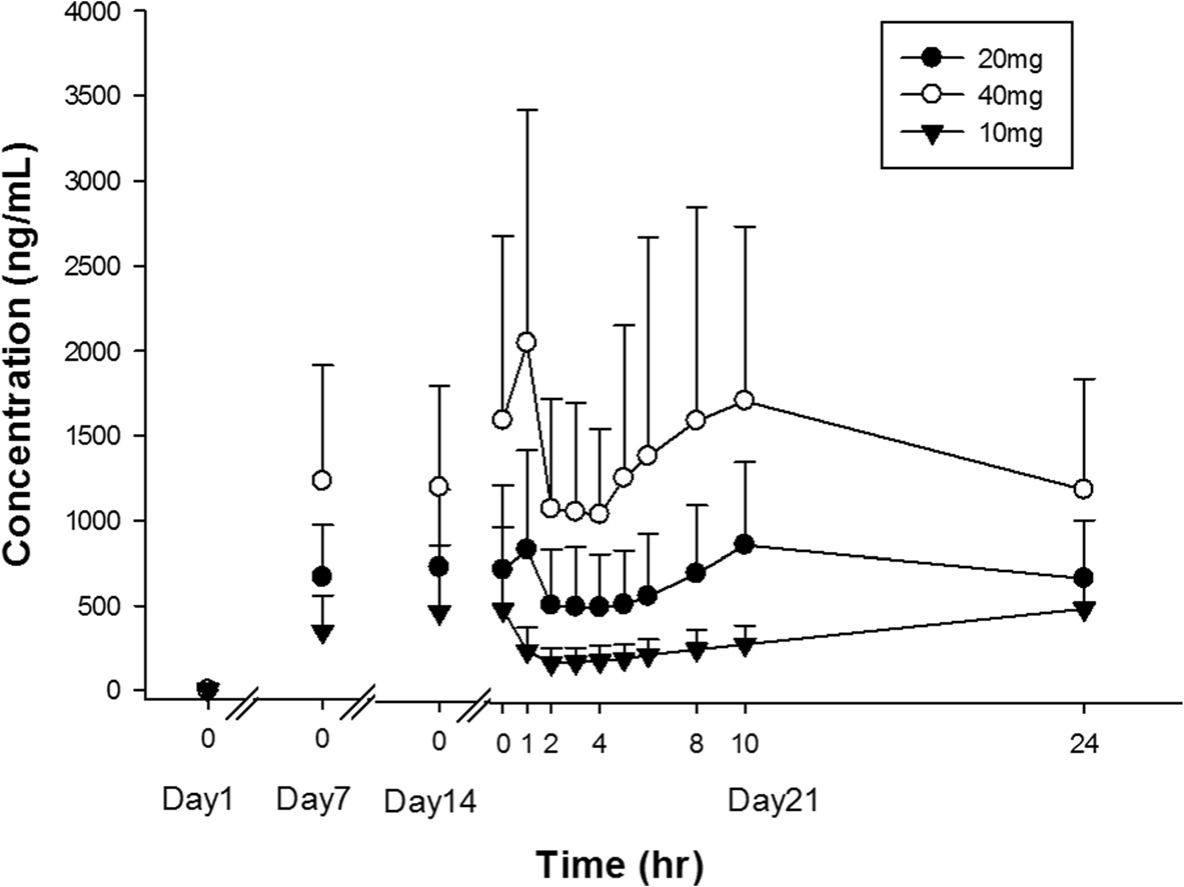
**Pradigastat plasma concentration-time profile.** Footnote: Data presented as mean ± standard deviation.

With once daily dosing one hour before breakfast, pradigastat appeared to achieve steady-state exposure by Day 14 as the dose normalized pre-dose concentrations appear to be comparable between Day 21 and Day 14. There was an approximately dose proportional increase in pradigastat exposure at studied doses. The maximal plasma concentration was achieved by a median of 1–10 hours at steady-state across the study dose range. Mean (SD) steady-state C_max_ and AUC_tau_ achieved for 10 mg, 20 mg, and 40 mg dose was 296 (111) ng/mL and 6,190 (2,660) ng*hr/mL, 950 (572) ng/mL and 18,000 (10,600) ng*hr/mL, and 2,170 (1,300) ng/mL and 39,900 (24,100) ng*hr/mL, respectively. Plasma concentration-time profile of pradigastat on Day 21 is shown in Figure 4.

### Safety and tolerability

Pradigastat was generally well tolerated in FCS patients at daily doses up to 40 mg for 3 weeks. There were no deaths or serious adverse events (SAEs) reported. None of the patients discontinued the treatment during the course of the study due to any adverse event (AE). A total of 66 AEs were reported by the six patients during the three treatment periods. All the AEs were mild to moderate in severity. AEs reported by the patients are shown in Table 4.

**Table 4:** **Adverse events reported by the patients during pradigastat treatment**

**Adverse events, n (%)**	**Treatment period 1**	**Treatment period 2**	**Treatment period 3**	**Total (n = 6)**
	**Pradigastat 20 mg (n = 6)**	**Pradigastat 40 mg (n = 6)**	**Pradigastat 10 mg (n = 4)**	
Diarrhea	4 (66.7)	5 (83.3)	3 (75.0)	6 (100.0)
Abdominal pain	3 (50.0)	2 (33.3)	1 (25.0)	4 (66.7)
Nausea	0 (0.0)	2 (33.3)	1 (25.0)	2 (33.3)
Flatulence	2 (33.3)	2 (33.3)	3 (75.0)	4 (66.7)
Abdominal pain upper	2 (33.3)	2 (33.3)	0 (0.0)	2 (33.3)
Fecal incontinence	1 (16.7)	0 (0.0)	0 (0.0)	1 (16.7)
Abdominal distension	1 (16.7)	0 (0.0)	0 (0.0)	1 (16.7)
Dizziness	0 (0.0)	1 (16.7)	0 (0.0)	1 (16.7)
Insomnia	1 (16.7)	1 (16.7)	0 (0.0)	1 (16.7)
Dyspepsia	1 (16.7)	0 (0.0)	0 (0.0)	1 (16.7)
Influenza	1 (16.7)	1 (16.7)	0 (0.0)	2 (33.3)

A patient with multiple occurrences of an adverse event is counted only once in a category during each treatment period.

The most common AEs reported were gastrointestinal events, including diarrhea, abdominal pain, and nausea. These AEs were usually mild and self-limited. There were no drug discontinuations or clinical evidence of volume depletion or electrolyte imbalance. The gastrointestinal AE profiles were similar across the different doses administered to the FCS patients. Diarrhea of moderate severity was reported for one day in only one patient each during the 20 and 40 mg dosing periods. There were no clinically significant abnormalities in hematology, blood chemistry, vital signs, or electrocardiogram (ECG) parameters.

## Discussion

Plasma TG levels are difficult to control in FCS patients, and as a result these patients are at high risk of developing recurrent pancreatitis [1,15]. Even strict adherence to very low fat diet is usually unsatisfactory [7]. Recently adeno-associated virus type I LPL gene therapy was approved in Europe for the treatment of FCS in a subset of patients with recurrent pancreatitis [9,16]. However, LPL gene therapy is costly and requires multiple intramuscular injections performed under general anesthesia. None of the currently available oral triglyceride lowering therapies are effective in FCS patients. We show here, for the first time, that the orally administered DGAT1 inhibitor pradigastat is associated with substantial reductions in plasma TG in FCS patients. This suggests that pradigastat may be an effective oral treatment for FCS. Pradigastat treatment over the course of 21 days was associated with up to a 70% reduction in fasting plasma TG compared to baseline. Reductions of plasma TGs of this magnitude are likely to be clinically meaningful in FCS patients, as plasma TG levels typically correlate with risk of acute pancreatitis and other complications such as xanthomas. With the limited sample size of this study, pradigastat appeared to achieve steady state exposure by day 14, which was maintained through day 21. At steady-state, exposure increased approximately dose-proportional between 10, 20 and 40 mg doses.

Furthermore, pradigastat appeared to be safe in FCS patients, with no treatment-induced clinically meaningful abnormalities in safety laboratories, ECGs, or physical findings. Pradigastat treatment was associated with mild and transient gastrointestinal AEs (most commonly diarrhea) but these did not lead to any drug discontinuation, or episodes of volume depletion. The very low fat diet recommended for FCS patients appears to improve the tolerability profile of DGAT1 inhibitors compared to other studies with higher dietary fat content [17]. Enrolled patients were all enthusiastic about their participation in the study, independent of the effect of pradigastat on fasting plasma TG concentration. Eruptive xanthomas disappeared in two participants with long history of very severe, hard-to-treat, xanthomatosis. A detailed review of the patients’ perspective is actually under investigation in this PoC study and in subsequent phase III sub-study.

Pradigastat has been shown to substantially reduce the rate of chylomicron-TG secretion into the lymphatic system following an oral fat load in animals [14]. Furthermore, pradigastat decreases the size and TG content of secreted chylomicrons. These effects are likely a result of direct DGAT1 inhibition of the absorptive enterocytes of the proximal small intestine, cells which absorb, re-esterify and secrete dietary fatty acids as chylomicron TG. Our findings that pradigastat reduces postprandial TGs, decreases chylomicron TG content, and reduces apoB48 levels in FCS patients are consistent with the hypothesis that DGAT1 inhibition reduces chylomicron secretion. This reduction of chylomicron-TG secretion may relatively off-load non-LPL TG clearance mechanisms in FCS patients, thus allowing previously circulating CM-TG to be cleared. In this study, the reduction of TG by pradigastat was almost entirely a result of the reduction in CM-TG, as VLDL-TG was mostly unchanged (Table 3). In FCS patients, almost all plasma TG is found in CM in both the fasted and fed state. This is different than in healthy subjects, where VLDL contains all the TG in the fasted state, while fed TG is generally split between CM and VLDL (and their remnants) in the postprandial state. Further studies will be needed to confirm that pradigastat decreases chylomicron-TG secretion in FCS, and to elucidate the mechanism by which fasting TG levels are decreased in these patients.

This exploratory study was conducted in a total of six FCS patients from a founder population who share the same underlying genetic defect in LPL. Although it is anticipated that pradigastat will have similar efficacy and tolerability in FCS patients with other defects in LPL or associated genes, this needs to be confirmed. Furthermore, the absence of a placebo control prevents a conclusive determination whether the end-of-treatment reduction in TG was due entirely to pradigastat treatment, or study design effects such as the low fat diet contributed to the observed TG lowering. Evidence from this and other studies suggest that the TG-lowering effects observed in this study were primarily mediated by pradigastat. Previous studies have shown that optimal out-patient low fat diet compliance in FCS patients can reduce fasting TG levels only to about 2000 mg/dL [1,18]. The one week low fat diet run-in in this present study reduced fasting TG levels to ~1900 mg/dL, suggesting that maximal diet effects had already occurred before pradigastat treatment began. Furthermore, despite the continued low fat diet given during the 21 days of treatment at the lowest and ineffective pradigastat dose (10 mg), there was no additional lowering in fasting TG level. This evidence suggests that pradigastat treatment (and not the low fat diet) mediated the continued drop in fasting TG below 2000 mg/dL during treatment at the 20 mg and 40 mg levels. A randomized, double-blind, placebo-controlled study will be needed to fully quantify the diet-independent TG-lowering effect of pradigastat in FCS patients.

## Conclusions

The DGAT1 inhibitor pradigastat, in addition to a very-low-fat diet, was associated with substantial reductions in fasting and postprandial TG in patients with FCS. In addition to reducing total TGs, pradigastat also reduced chylomicron TG content, and apoB48 levels. Pradigastat was safe and generally well tolerated in this FCS patients in this study. Based on the encouraging results of this exploratory study, pradigastat is currently being studied in a randomized, double-blind, placebo-controlled, phase III study in FCS patients (Novartis Pharmaceuticals. A randomized, double-blind, placebo controlled study to assess efficacy, safety and tolerability of LCQ908 in subjects with familial chylomicronemia syndrome. ClinicalTrials.gov, NCT01514461).

## Methods

The study was conducted from May 2010 to May 2011, in compliance with Good Clinical Practice as outlined in the principles of the Declaration of Helsinki. The study was approved by the institutional review board/independent ethics committee and written informed consent was obtained from all the participating patients. The study was registered with ClinicalTrials.gov (NCT01146522).

### Patients

FCS patients aged 18–75 years not on any lipid-lowering medications for ≥8 weeks prior to enrolment were eligible for the study. All subjects were recruited at a single clinical site in Quebec, Canada, where there is a population of FCS patients due to a founder effect [18]. To be included, patients had to meet at least two of the following criteria: fasting TG ≥890 mg/dL (>10 mmol/L); post-heparin plasma LPL activity ≤20% of normal; LPL mass >5% and/or confirmed homozygote or compound heterozygote mutations in LPL gene (null alleles) with LPL mass >5% and LPL activity ≤20%. Pregnant/nursing women and patients with uncontrolled diabetes or an active pancreatitis episode within 1 month of enrollment were excluded. No medication other than the study drug was allowed from the first dosing until all of the study evaluations were completed, except for medication that may have been required to treat AEs or preexisting co-morbidities.

### Study design and treatments

**Figure 5:**
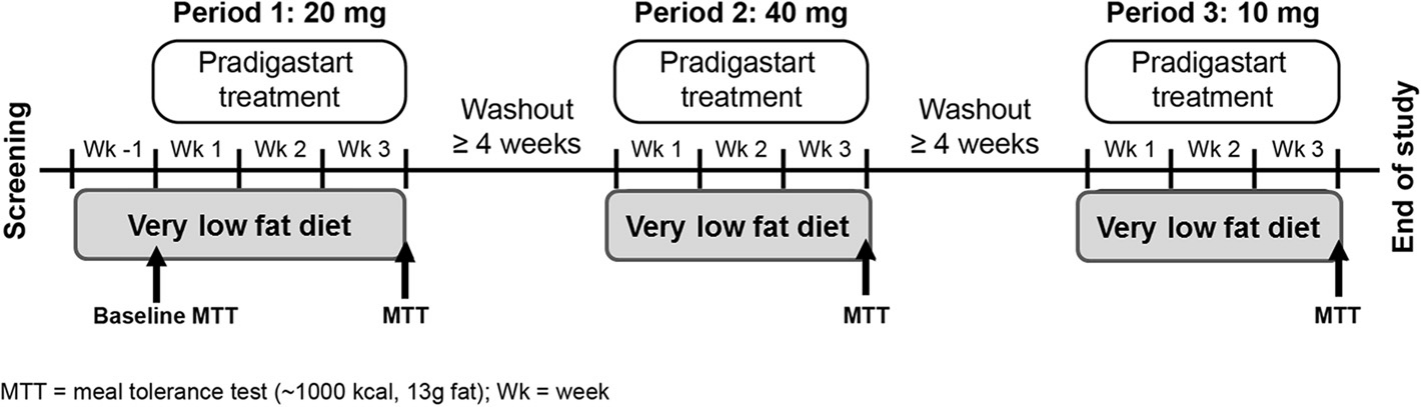
**Study design.**

This was an open-label, three-period, sequential treatment study, with enrolled patients undergoing a 1-week run-in period with low-fat (~20%) diet to stabilize TG levels (Figure 5). Following this, pradigastat was administered orally once daily for three weeks in each of the three periods in a non-randomized sequence at 20 (period 1), 40 (period 2), and 10 mg (period 3) doses. During the treatment periods, patients remained on the same low-fat diet. There was a drug washout period of at least four weeks between each treatment period, during which time the patients also observed a low-fat diet.

### Study procedures

Following confirmation of eligibility, patients began the study on Day-7 when the low-fat diet was instituted to stabilize TG levels. On Days −3 and −2, patients were provided with frozen standardized low-fat meals prepared by a Chef in collaboration with a trained dietician to ensure dietary compliance. In the morning of Day −1, following an overnight fast, blood samples were collected to assess a baseline fasting lipid profile, after which a MTT was performed. A test meal with approximately 972 kcal, 13.5 g of lipids (≈13% of calories), 155 g of carbohydrates (≈64%), and 56 g of proteins (≈23%) was administered, and blood samples were collected frequently through 10 hours (pre-dose, 1, 2, 3, 4, 5, 6, 8, 10). Patients were discharged thereafter and asked to return to the study site next morning (Day 1) for blood collection 24-hour post MTT. The baseline MTT occurred only at the beginning of treatment period 1.

From Day 1 to Day 21 of period 1, all patients received pradigastat 20 mg orally once daily approximately 1 hour before breakfast on an outpatient basis. Fasting blood samples were collected on Days 1, 7, 14, and 21 pre-dose for assessment of plasma lipids. Safety and compliance assessments were carried out at weekly clinic visits and by telephone monitoring at select times in between the visits. Patients filled in a meal diary during the baseline and all subsequent study periods to confirm compliance with the diet. On Day 21, an on-treatment MTT was performed, identical to the one done on Day −1. Following a 24 hour post-MTT blood draw on the morning of Day 22, patients returned to their homes, with instructions to continue the low-fat diet.

Following a washout period of at least four weeks, patients returned to the clinical site for the start of period 2, where they received pradigastat 40 mg a day for 21 days. As with period 1, blood samples were taken on Days 1, 7, 14, and 21, and another MTT was administered on day 21. Following another washout period of at least 4 weeks, patients returned for a third and final treatment period with 10 mg pradigastat. As with the other periods, fasting blood samples were taken throughout the period, and a MTT was administered on Day 21. Following the final 24 hour post-MTT blood sample, patients returned to their home. An end of study visit was performed at least 14 days after the final treatment period ended. During washout periods, patients did not receive study medication, thus allowing pradigastat to be cleared from the plasma before the next treatment period started. Additionally, patients were instructed to maintain their usual low fat diet during the washout period.

### TG and other laboratory parameters’ assessments

Blood samples were analyzed for TG using an enzymatic hydrolysis method utilizing a Synchron LX® System (Beckman Coulter, Inc., Brea, CA, USA). Plasma was fractionated at the Ecogene-21 lab into different lipoprotein fractions by sequential steps of density gradient ultracentrifugation. The first ultracentrifugation step separated chylomicron (CM) particles (Sf > 400) from the rest of the plasma. Following removal of the CM layer, additional ultracentrifugation steps separated the plasma into sf < 400 fractions: very-low density lipoprotein, low-density lipoprotein, and high-density lipoprotein. Each fraction was collected by aspiration from the top of the centrifuge tube. Each of the four lipoprotein fractions (CM, VLDL, LDL and HDL) had the following measurements were taken: TG and cholesterol (using standard colorimetric methods) and Apoproteins by nephelometry.

### Pharmacokinetic assessments

Blood samples (2 mL) for pharmacokinetic evaluation were collected by either direct venipuncture or an indwelling cannula into EDTA-containing tubes on Days 1, 7 and 14 pre-dose and at the following time points on Day 21: pre-dose, 1, 2, 3, 4, 5, 6, 8, 10 and 24 hours post-dose. The pharmacokinetic parameters were determined using a non-compartmental method (s) that included area under the concentration-time curve from time zero to the end of dosing interval (0-24 h) (AUC_tau_), highest concentration observed during a dosing interval at steady-state (C_max,ss_) and the time to reach maximum plasma drug concentration after single dose administration (T_max_).

### Safety assessments

Safety assessments during the study included physical examination; ECG; vital signs; standard clinical laboratory evaluations including hematology, blood chemistry, and urinalysis; and AE and SAE monitoring. AEs were evaluated by recording their onset, duration, severity, relation to the therapy, and treatment required.

### Statistical analysis

A dose comparison was carried out for fasting TG data, which was analyzed using a linear mixed effect model for repeated measurements. The model included treatment, time, and treatment by time interaction as factors; baseline as a covariate; and subject as a random effect. Postprandial peak and AUC TG were analyzed for dose comparison by a linear mixed effect model, with treatment and baseline values as fixed effects and subject as a random effect; however, the pharmacokinetic parameters were analyzed on Day 21 using an ANOVA model with dose level as a factor, and subject as a random effect. Estimates of the treatment effect of different dose levels, together with 90% CI were obtained. Log-transformation was applied prior to the analysis and the results were back transformed and reported in the original scale. All the above analyses were repeated for secondary end points. Missing measurements for AUC were imputed by linear interpolation only if two adjacent time points had observed data and was set to missing otherwise. Missing measurements at the end of the time interval were imputed from the previous time point.

## Abbreviations

AE: Adverse event
ApoB48: Apolipoprotein B48
AUC: Area under the curve
AUC_0–9_: Area under the curve over the 9 hours
CI: Confidence intervals
CM: Chylomicron
DGAT1: Diacylglycerol acyltransferase-1
ECG: Electrocardiogram
FCS: Familial chylomicronemia syndrome
LDL: Low-density lipoprotein
LPL: Lipoprotein lipase
MTT: Meal tolerance test
SAE: Serious adverse event
SD: Standard deviation
TG: Triglyceride
UGT: UDP-glucuronosyltransferase
VLDL: Very-low-density lipoprotein
